# Incidence and risk factors for surgical site infection following volar locking plating (VLP) of unstable distal radius fracture (DRF)

**DOI:** 10.1186/s13018-022-03440-7

**Published:** 2022-12-19

**Authors:** Hongyu Meng, Bin Xu, Yi Xu, Haiyun Niu, Ning Liu

**Affiliations:** 1 Department of Orthopaedic Surgery, The 3rd Hospital of Hebei Medical University, No. 139 Ziqiang Road, Shijiazhuang, 050051 Hebei People’s Republic of China; 2 Key Laboratory of Biomechanics of Hebei Province, Shijiazhuang, 050051 Hebei People’s Republic of China; 3 Orthopaedic Institution of Hebei Province, Shijiazhuang, 050051 Hebei People’s Republic of China

**Keywords:** Distal radius fracture, Surgical site infection, Risk factor, Perioperative condition optimization

## Abstract

**Purpose:**

Volar locking plating (VLP) is the mainstay of treatment for distal radius fracture (DRF) but may be compromised by postoperative surgical site infection (SSI). This study aimed to identify the incidence and the risk factors for SSI following VLP of DRF.

**Methods:**

This retrospective study identified consecutive patients who underwent VLP for closed unstable DRFs in our institution between January 2015 and June 2021. Postoperative SSI was identified by inquiring the medical records, the follow-up records or the readmission medical records for treatment of SSI. The potential factors for SSI were extracted from the medical records. Univariate and multivariate logistic regression analyses were performed to identify the independent factors.

**Results:**

There were 930 patients included, and 34 had an SSI, representing an incidence of 3.7% (95% CI 2.4–4.9%). Patients with an SSI had threefold extended hospitalization stay (44.1 ± 38.2 versus 14.4 ± 12.5 days) as did those without. In univariate analysis, 18 variables were tested to be statistically different between SSI and non-SSI group. In multivariate analysis, 6 factors were identified as independently associated with SSI, including sex (male vs. female, OR 3.5, *p* = 0.014), ASA (III and IV vs. I, OR 3.2, *p* = 0.031), smoking (yes vs. no, OR 2.4, *p* = 0.015), bone grafting (OR 4.0, *p* = 0.007), surgeon volume (low vs. high, OR 2.7, *p* 0.011) and operation at night-time (vs. day-time, OR 7.8, *p* < 0.001).

**Conclusion:**

The postoperative SSI of VLP of DRF was not uncommon, and the factors identified in this study, especially those modifiable, would help identify individual SSI risk, target clinical surveillance and inform patient counseling.

## Introduction

Distal radius fracture (DRF) is among the most common fractures in emergency or orthoapedic department, with bimodal peaks in young patients less than 18 years old and in the elderly aged 65 years or older. Open reduction and volar locking plating (VLP) has become the mainstay of treatment for unstable DRF since being introduced into practice, due to optimal biomechanical performance and clinical and radiographic results [1, 2]. However, as every other surgical modality, the surgical results of VLP are affected by the postoperative surgical site infection (SSI), a relatively uncommon but devastating complication. In particular, the deep SSI generally required substantially prolonged antibiotic course and even the secondary surgery for debridement, hardware exchange or removal. In addition, the increased healthcare cost caused by extended hospitalization stay for treatment of SSI was also a concern.

Studies characterizing the SSI after VLP of DRF reported the greatly variable incidence rate, ranging from 0 to 5.6%, primarily based on the heterogeneous population and inconsistent definitions of SSI [3–5]. In 2015, a meta-analysis of 7 randomized controlled trials comprising 875 patients undergoing VLP versus percutaneous pinning of displaced DRFs reported an incidence rate of SSI of 3.2% [6]. Similarly, regarding the risk factors for SSI, results were inconclusive and occasionally even conflicting, primarily due to the heterogeneous population, relatively small sample size, or inclusion of limited number of variables for adjustment [7–9]. However, to our best knowledge, none of these studies were specifically designed to address the SSI after VLP, and thus these results might be less applicable.

Understanding of the incidence rate of SSI and knowledge of the related risk factors will provide a plausible approach for prevention via optimizing medical conditions, especially those modifiable ones. More importantly, prevention, compared to treatment of SSI itself, is far more cost-effective. Thus, we designed this study to examine the incidence and risk factors for SSI after VLP of DRFs.

## Methods

### Data resource and inclusion and exclusion criteria

This was a retrospective study, enrolling patients who presented with a closed DRF and definitely underwent open reduction and VLP procedure in the Third Hospital of Hebei Medical University between January 2015 and June 2021. The study protocol was approved by the ethics committee of our institution, which waived the need for informed consent because of the deidentified data used, and it was conducted in accordance with the Declaration of Helsinki.

Patients who were included in this study met the following criteria: age of 18 years or older, closed DRF treated by VLP within 21 days of injury, complete data recorded in the electronic medical records and a minimum of 12-month follow-up assessments. Patients who were excluded if they had an old fracture (> 21 days after injury), bilateral DRFs, other fractures in the affected upper limb, pathological or metastatic fracture, polytrauma, other operative procedures (conservation, dorsal plating, percutaneous fixation, external fixation et al.), existing wound infection before operation, psychotropic drug abuse or glucocorticoid dependence, missing data for variables of interest, or no follow-up assessment data.

### Operative procedure and postoperative management

The modifiable volar Henry approach was applied the VLP procedure. Prophylactic antibiotics (e.g., cefazolin, most commonly, or vancomycin in cases of allergy) in single dose were rousingly administered 30 min prior to skin incision or tourniquet inflation. The pronator quadratus muscle was cut off or not, repaired or not if cut off, implant type, number of screws placed, or postoperative drainage was based on the surgeon’s preference. Postoperatively, the wrist was immobilized in a below-elbow cast for 2–4 weeks, based on bone quality and fracture severity. Active finger motion was started at postoperative day 1, and sutures were removed on day 14. Follow-up was performed at 2 weeks, 1, 3, 6 and 12 months postoperatively to evaluate the radiographic bone union, wound union, functional recovery or any potential complications.

### Definition of categorization of SSI

SSI was defined in accordance with the criteria proposed by Centers of Disease Control and Prevention in 2017 [10]. SSI was categorized as surficial or deep. A superficial SSI refers to an infection that involves skin or superficial fascia within 1 month of operation, leading to pain, tenderness, redness and heat over the site, and generally resolves with a course of oral antibiotics or local wound care without operative intervention. A deep SSI refers to an infection that extends deep fascia or muscle, occurring within 12 months after the operation. Symptoms or signs as purulent drainage, abscess formation, spontaneous incisional dehiscence plate fistula and systematic elevated body temperature (≥ 38°) and chill were considered, which generally require intravenous antibiotics, wound debridement or even plate removement. Laboratory culture results of the samples obtained from superficial swabs or operated deep tissues were used for auxiliary diagnosis, but not dependent on.

### Data collection

Two investigators (H.M. N.L.) were responsible for data collection. Relevant data were extracted from the patients' hospitalization medical records, including demographics characteristics (age, sex, occupation, living place), lifestyles (smoking, alcohol drinking), comorbidities (body mass index (BMI), hypertension, diabetes, heart disease, cerebrovascular disease, chronic obstructive pulmonary disease, and renal insufficiency), injury (injury mechanism, affected side (left or right; dominant or non-dominant), fracture type per Arbeitsgemeinschaft für Osteosynthesefragen (AO) classification, surgery (anesthesia mode, American Society of Anesthesiologists (ASA) classification), surgical timing (day or night-time), surgical duration, blood loss, bone grafting, allogenic blood transfusion, drainage use). The laboratory indexes, i.e., serum albumin level, count of red blood cell (RBC), hemoglobin, white blood cell (WBC), neutrophils, lymphocytes and platelet, and sodium concentration that were determined a priority in orthopedic or other surgeries [11–14] were also extracted.

Renal insufficiency was defined as a serum creatinine of 1.5 mg/L or higher [15]. Low-energy fracture was defined as a fracture caused by a fall from a standing height, and high-energy fracture by fall from a height, traffic accident, or mechanical impaction or punch. The smoking or alcohol drinking status was determined according to the documented in the medical record.

### Statistical analysis

SSI or non-SSI group was defined according to the presence or absence of postoperative SSI. Statistical descriptions for continuous variables were expressed with mean ± standard deviation (SD) and for categorical variables with frequency and percentage (%). The normality of continuous variable was explored by Shapiro–Wilk test. For data normally distributed, the between-group difference was detected by Student *t* test, otherwise, by Mann–Whitney *U* test. Categorical data were compared by using Chi-square test or Fisher’ exact test (in cases of more than 25% of the expected were under 5), as appropriate.

Variables tested with *p* < 0.10 in the univariate analyses were further entered into the multivariate logistic regression analysis to identify their independent effect on SSI. In the multivariate model, the well-established variables in literature relating to SSI (diabetes, hypoalbuminemia) and demographics (age and sex) regardless of statistical significance would be included for adjustment. The “enter” method was used. The goodness of fit of the multivariate model was evaluated by Hosmer–Lemeshow test, and the result was quantified by the adjusted Nagelkerke *R*^2^, with < 0.750 suggestive of an acceptable result [16]. The magnitude of association between the variable and SSI was indicated by odds ratio (OR) with 95% confidential interval (CI). The statistical significance level was set as *p* < 0.05. SPSS software 26.0 (IBM corporation, New York, USA) was used for all statistical analyses.

## Results

This study included 930 patients, 482 male and 448 female patients, with an average age of 48.0 ± 14.5 years. A total of 57 orthopedics surgeons performed the all 930 procedures, with each with a median of 14 cases (range, 1 to 68 cases) during the study period. According to the number of procedures performed from low to high, twenty-two orthopedic surgeons performed the 194 (about 20%) and were considered as low-volume, and the remaining 35 who performed about 80% (736) procedures as high-volume.

There were 34 (3.7%) cases of SSI, including 21 (2.3%) superficial and 13 (1.4%) deep SSIs. Patients with an SSI had threefold extended hospitalization stay (44.1 ± 38.2 versus 14.4 ± 12.5 days) as did those without (*p* < 0.001). Univariate analyses showed SSI and non-SSI patients significantly differed in terms of sex, age in continuous variable, ASA classification, anesthesia mode, bone grafting, operative time, allogeneic transfusion, operation timing, serum albumin, sodium concentration, WBC count, neutrophil count, RBC count, hemoglobin level, surgeon volume and hematocrit (all *p* < 0.05). (Table 1).

**Table 1:** Univariate analysis of variables between SSI and non-SSI group

Variables	Number (%) of patients without SSI (*n* = 896)	Number (%) of patients with SSI (*n* = 34)	*p*
**Age (years)**	48.1 ± 14.5	44.1 ± 13.5	0.115
18–64	778 (86.8)	33 (97.1)	0.080
≥ 65	118 (13.2)	1 (2.9)	
**Sex (males)**	456 (50.9)	28 (82.4)	< 0.001
**Living place (rural)**	662 (73.9)	24 (70.6)	0.668
**Occupation (manual work)**	691 (77.1)	27 (79.4)	0.755
**BMI (kg/m**^**2**^**)**	25.2 ± 3.5	25.0 ± 3.0	0.674
≥ 28	178 (19.9)	6 (17.6)	0.750
**Hypertension**	140 (15.6)	6 (17.6)	0.750
**Diabetes mellitus**	158 (17.6)	5 (14.7)	0.659
**Cerebrovascular disease**	49 (5.5)	2 (5.9)	0.917
**COPD**	26 (2.9)	2 (5.9)	0.318
**Renal insufficiency**	16 (1.8)	1 (3.6)	0.472
**Current smoking**	186 (20.8)	13 (38.2)	0.015
**Alcohol drinking**	322 (35.9)	14 (41.2)	0.533
**Injury mechanism (high-energy)**	135 (15.1)	9 (26.5)	0.071
**Fracture type (based on AO classification system)**			0.806
A	176 (19.6)	7 (20.6)	
B	113 (12.6)	3 (8.8)	
C	607 (67.7)	24 (70.6)	
**Time from injury to operation (days)**	4.4 ± 4.0	5.0 ± 4.9	0.366
** < 7**	740 (82.6)	24 (70.6)	0.073
≥ **7**	156 (17.4)	10 (29.4)	
**Surgical duration (minutes)**	128.5 ± 66.4	192.1 ± 103.2	0.001
≥ 180	177 (19.8)	17 (50.0)	< 0.001
**Operation timing**			< 0.001
Day-time	870 (97.1)	28 (82.4)	
Night-time	26 (2.9)	6 (17.6)	
**ASA classification**			0.001
I	174 (19.4)	3 (8.8)	
II	631 (70.4)	21 (61.8)	
III–IV	91 (10.2)	10 (29.4)	
**Anesthesia mode**			< 0.001
Regional	706 (78.8)	18 (52.9)	
General	190 (21.2)	16 (41.7)	
**Surgeon volume (low)**	180 (20.1)	14 (41.2)	0.003
**Bong grafting**	65 (7.3)	6 (17.6)	0.025
**Blood loss (ml)**	144.5 ± 212.6	434.6 ± 385.9	0.007
≥ 400	92 (10.3)	12 (35.3)	< 0.001
**Allogeneic blood transfusion**	49 (5.5)	8 (23.5)	< 0.001
**Albumin level (**g/L**)**	41.6 ± 5.2	37.3 ± 6.3	< 0.001
< 35	94 (10.5)	13 (38.2)	< 0.001
**Sodium concentration (mmol/L)**	139.7 ± 3.2	137.0 ± 3.7	< 0.001
< 135	60 (6.7)	8 (23.5)	< 0.001
WBC (*10^**9**^** /L)**	8.3 ± 2.6	10.2 ± 4.3	< 0.001
> 10	195 (21.8)	14 (41.2)	0.008
Neutrophil count (*10^**9**^**/L)**	5.8 ± 2.4	7.6 ± 4.0	0.014
> 6.3	328 (36.6)	20 (58.8)	0.009
**Lymphocyte count (10**^**9**^**/L)**	1.7 ± 0.6	1.5 ± 0.5	0.043
< 1.8	144 (16.1)	6 (17.6)	0.806
**RBC (10**^**12**^**/L)**	4.3 ± 0.6	4.0 ± 0.7	0.030
< lower limit	86 (9.6)	12 (35.3)	< 0.001
**Hemoglobin (g/L)**	131.1 ± 17.8	121.0 ± 20.5	0.001
< lower limit	108 (12.1)	13 (38.2)	< 0.001
**Hematocrit (%)**	39.0 ± 5.2	36.1 ± 6.0	0.001
< lower limit	173 (19.3)	17 (50.0)	< 0.001

BMI, body mass index; COPD, chronic obstructive pulmonary disease; ASA, American Society of Anesthesiologists; WBC, white blood cell; RBC, red blood cell, reference range: females, 3.5–5.0*10^12^/L; males, 4.0–5.5*10^12^/L; reference range for hemoglobin: Females, 110-150 g/L and males, 120-160 g/L; reference range for hematocrit: Females, 35%-45%; males, 40%-50%

The multivariate analysis showed that 6 variables were identified as independent risk factors associated with development of SSI, including sex (male vs. female, OR 3.5, *p* = 0.014), ASA (III and IV vs. I, OR 3.2, *p* = 0.031), smoking (yes vs. no, OR 2.4, *p* = 0.015), bone grafting (OR 4.0, *p* = 0.007), surgeon volume (low vs. high, OR 2.7, *p* = 0.011) and operation timing (night vs. day-time, OR 7.8, *p* < 0.001) (Table 2). The Hosmer—Lemeshow test showed acceptable goodness of fit, with *p* value of 0.301 and the adjusted Nagelkerke *R*^2^ value of 0.456.

**Table 2:** Multivariate logistic analysis of variables independently associated with SSI after VLP of DRFs

Variables	OR and 95% CI	*p*
Sex (male vs. female)	3.5 (1.3–9.8)	0.014
ASA		
I	Reference	
II	1.1 (0.3–3.8)	0.874
III and IV	3.2 (1.1–14.8)	0.031
Smoking (yes vs. no)	2.4 (1.2–4.8)	0.015
Bone grafting	4.0 (1.4–11.6)	0.007
Surgeon volume (low vs. high)	2.7 (1.4–7.7)	0.011
Operation timing (night vs. day-time)	7.8 (2.6–23.5)	< 0.001

## Discussion

This study used a large sample of patients to evaluate the incidence and risk factors for the SSI after VLP of DRFs. The findings showed the incidence of SSI was 3.7%, and 6 factors were identified to be significantly associated with SSI, including male sex, ASA (III and IV versus I), smoking, bone grafting, low surgeon volume, and operation at nighttime (vs. daytime).

The incidence rate of SSI following VLP of DRF was found to be 3.7%, within the range described in literature. Two large studies using national database reported the far lower incidence rates of SSI, the one was 0.9% within 180 days of fixation (1.3% for percutaneous and 0.8% for open fixation) in 87,169 DRF patients [17], and the other was 0.3% within 90 days in patients who underwent the open reduction and internal fixation for DRF [7]. In another nation-wide study of 31,807 adult patients undergoing plating of DRFs, incidence rate of SSI was 5% [9]. However, these studies did not separate the VLP procedure and even in Mahmood et al.’ study SSIs resolved by oral antibiotics were not counted [7], thus likely not reflecting the true SSI rate after VLP. Recently, Wang et al. [18] reported a 6.7% rate of SSI within 12 months after VLP. In their study, a certain proportion of open fracture (16.4%, 103/627) was included and 40% (17/25) of SSIs occurred in open fractures, largely explaining the so high SSI rate [18]. In contrast, we only included the closed DRFs. These varied figures might reflect the differences in study population, study design, definition of SSI and the follow-up period among studies, also the hospital and surgeon level.

The male sex, higher ASA grade and current smoking status have been well established in relation to the risk of wound complications in orthopedic trauma literature [19–22], which were re-confirmed in this study of VLP for treatment of DRFs. Thus, for these well-established risk factors that patients who were involved, treating surgeons should keep in mind the increased risk of SSI. Quitting smoking should be encouraged immediately upon patients’ admission, because even cessation of smoking for 5 days preoperatively would have a positive effect on wound healing [23] and 4-week abstinence from smoking would produce a similar benefit in reduction of wound infections as those who never smoke [24].

The role of night-time operation in postoperative complications was conflicting across different surgeries [25–27], and in this study patients operated on at night-time had a strongest risk of SSI (OR 7.8) when compared to those operated on at day-time. The most likely explanation was that the surgeon fatigue, decreased availability of support staff (e.g., medical, anesthesia or on-call operating room staff), and logistic factors (hospital resources) caused a delay with instrumentation and an extended surgical time [27]. It was estimated that surgeons who had overnight shift experienced 20% more errors and 14% longer time on laparoscopy simulator [28]. Another explanation might be related to property of night-time procedures, which were generally emergent operative procedures for severe fractures that were caused by high-energy trauma and were expected to cause severe swelling soon to delay the subsequent surgery; thus, for these cases, the relatively poor soft tissue and the insufficient preoperative preparation inherently carried a higher risk of postoperative wound complication.

The benefits of bone grafting for radiographic and functional outcomes were challenged and, for complications, were conflicting [29, 30]. In this study, we did not evaluate the outcome measures, but found that bone grafting (autogenous and allogenous bone grafts combined into a category) was associated with a fourfold increased risk of SSI. For allogenous bone grafting, the weak antibacterial property, the risk of bio-incompatibility and immunological rejection combined with the poor blood supply at the surgical site would play a major role [31]. For autogenous bone grafting, the graft-harvesting procedure itself would induce a significantly extended surgical duration and thus the chance to expose bacteria. Additionally, the increased blood loss would partly impair the systemic ability of the host to resist bacterial infection [32].

The VLP procedure is technically demanding and needs a steep learn curve; with increasing experience, surgeons will gain greater knowledge and skill to allow them to avoid pitfalls, thus reducing the possible complications. In a prior study of early complication of VLP in relation to surgeon experience, the first operated cohort of 30 patients experienced significantly more complications (37% vs. 17%) than the later treated cohort of 62 patients [33]. In this study, number of accumulated VLP procedures for DRF for one surgeon since practice was not captured; instead, surgeon case volume during the study period was used. Indeed, in 2015, Tang et al. [34] stated the importance of levels of expertise of surgeons in reporting outcomes of treatment, and classified them into 5 levels/categories, and an experienced surgeon was defined as “a surgeon who has obtained appreciable experience in use of the relevant technique(s), having practiced as a specialist over a longer period (typically > 5 years)”. From this definition, the effect of less experience on SSI might have been weakened by our study, because about 1/3 low-volume surgeons are experienced.

Several limitations to this study should be noted. First, the retrospective study design had its inherent limitation in data collection, especially the patients’ self-reported comorbidities might not reflect the true case. Additionally, we could not quantify the volume of smoking status or alcohol drinking, which have demonstratable dose-dependent effect on wound complications. Second, SSI cases were confirmed by inquiring the medical records of index hospitalization for VLP of DRF or of readmission for treatment of SSI, and the routine outpatients visit postoperatively. Postoperative SSIs that were treated in other institutions or minor SSIs that resolved without readmission might have been missed in this study, thus creating an underestimation of actual incidence. Third, the severity of the skin and soft tissue around the fracture or surgical site would contribute a role in SSI development, but was not measured and not included for adjustment. Fourth, the single-center design of this study in a tertiary referral and university hospital might make the results less generalizable to populations treated in different level institutions.

In conclusion, the incidence rate of SSI following VLP of DRFs was 3.7% and 6 variables were identified, including male sex, greater ASA classification (III and IV), current smoking, bone grafting, low surgeon volume, and operation at nighttime (vs. daytime). These results would help identify individual SSI risk, target clinical surveillance and inform patient counseling. Smoking cessation should be encouraged immediately after admission, and when possible, VLP procedure is better performed under the supervision of an experienced surgeon at day-time, and bone graft can be decreased for use.

## Data Availability

In accordance with institutional policy for patients' medical records, data used in this study are not open to public, but will be available upon motivated request to the corresponding author for purpose of scientific research.

## Abbreviations

DRF: Distal radius fracture
VLP: Volar locking plating
SSI: Surgical site infection
BMI: Body mass index
ASA: American Society of Anesthesiologists
COPD: Chronic obstructive pulmonary disease
SD: Standard deviation
OR: Odds ratio
CI: Confidential interval
WBC: White blood cell
RBC: Red blood cell
AO: Arbeitsgemeinschaft für Osteosynthesefragen
